# Supplementary material for: The role of Pitx2 and Pitx3 in muscle stem cells gives new insights into P38α MAP kinase and redox regulation of muscle regeneration
Source: eLife. 2018 Aug 14;7:e32991. doi: 10.7554/eLife.32991 (PMC6191287; doi:10.7554/eLife.32991)
Supplement: Supplementary file 1. [file elife-32991-supp1.docx]

| **Mouse lines** | **Mutation** | **Experiments** |
| --- | --- | --- |
| *Pax3^GFP/+^* | Constitutive | Satellite cell purification for primary culture or for analysis during regeneration |
| *Pitx3^+/-^* | Constitutive | Regeneration experiments |
| *Pitx3^-/-^* | Constitutive | Regeneration experiments |
| *Pitx3^+/-^:Pax3^GFP/+^* | Constitutive | Satellite cell purification for primary culture or for analysis during regeneration |
| *Pitx3^-/-^:Pax3^GFP/+^* | Constitutive | Satellite cell purification for primary culture or for analysis during regeneration |
| *Pitx2^flox/+^:R26R^Cre-ERT2/+^* | Inducible | Satellite cell purification for analysis during regeneration |
| *Pitx2^flox/flox^:R26R^Cre-ERT2/+^* | Inducible | Satellite cell purification for analysis during regeneration |
| *Pitx2^flox/+^:Pitx3^+/-^:Pax7^Cre-ERT2/+^* | Inducible | Regeneration experiments |
| *Pitx2^flox/+^:Pitx3^+/-^:R26R^Cre-ERT2/+^:Pax3^GFP/+^* | Inducible | Regeneration experiments |
| *Pitx2^flox/flox^:Pitx3^flox/-^:R26R^Cre-ERT2/+^:Pax3^GFP/+^* | Inducible | Satellite cell purification for primary culture or for analysis during regeneration |
| *Pitx2^flox/+^:Pitx3^+/-^:Pax7^Cre-ERT2/+^:mdx* | Inducible | Satellite cell purification for primary culture or for analysis during regeneration |
| *Pitx2^flox/flox^:Pitx3^flox/-^:Pax7^Cre-ERT2/+^:mdx* | Inducible | Homeostasis |
| *Rag2^-/-^:Il2Rb^-/-^:dmd/dmd* | Constitutive | Homeostasis |
