# Supplementary material for: The role of Pitx2 and Pitx3 in muscle stem cells gives new insights into P38α MAP kinase and redox regulation of muscle regeneration
Source: eLife. 2018 Aug 14;7:e32991. doi: 10.7554/eLife.32991 (PMC6191287; doi:10.7554/eLife.32991)
Supplement: Supplementary file 14 [file elife-32991-supp2.docx]

Primer sequences used for RT-qPCR				
**Function**	**Gene**	**Name**	**Forward primer**	**Reverse Primer**
Mitochondrial genes and metabolism	*Ucp2*	Uncoupling protein 2	CCAGCCTACAGATGTGGTAAAG	CTCTCGTGCAATGGTCTTGT
	Hprt	Hypoxanthine guanine phosphoribosyl transferase	CGAGATGTCATGAAGGAGATGG	AGCAGGTCAGCAAAGAACTTA
Quantification of mitochondrial DNA	*COI*	Cytochrome C oxidase subunit I	TGCTAGCCGCAGGCATTAC	GGTGCCCAAAGAATCAGAAC
	NDUFV1	NADH:Ubiquinone Oxidoreductase Core Subunit V1	CTTCCCCACTGGCCTCAAG	CCAAAACCCAGTGATCCAGC
Oxidative stress	*SelX*	Selenoprotein X	GAAACCATCCACCCAGACAG	TCTTGATTGTCCCCGCTTG
	SelM	Selenoprotein M	GTCCACCGTGTATGCCTTCT	CTCCTGGTGTCCGAACTGAT
	Mgst2a	Microsomal glutathione S-transferase 2 alpha	CGCCCGTCACAAGTATTTCTG	AAAGTCCAGGTATTCGTTCAGG
	Gstz	Glutathione S-transferase zeta	ACCTCCTGCAAGAAGAGCTG	GCTGGGTTGGTCCGATACTA
	Hspb2	Heat Shock Protein Binding 2	CTCCCGTGGTGATTCCATAAA	TCAAGGGACCAAACCATACAC
Senescence	*IGFBP5*	Insulin Growth Factor Binding Protein 5	GCAGGGAACCTGGAGAATAAA	TCCCAAGCCTTGTCTGTTTAG
	Ccl2	Chemokine (C-C motif) ligand 2	CTCACCTGCTGCTACTCATTC	ACTACAGCTTCTTTGGGACAC
	Ccl5	Chemokine (C-C motif) ligand 5	CCAATCTTGCAGTCGTGTTTG	ACCCTCTATCCTAGCTCATCTC
	Ifitm1	Mouse interferon induced transmembrane protein 1	GGTCTGGTCCCTGTTCAATAC	GTCGTATCACCCACCATCTTC
	mdm2	Mouse transformed mouse 3T3 cell double minute 2	AGTCTCTGGACTCGGAAGATTA	CTGTATCGCTTTCTCCTGTCTG
Transcription factors	*Myog*	Myogenin	CAACCAGGAGGAGCGCGATCTCCG	AGGCGCTGTGGGAGTTGCATTCACT
	Nrf1	Nuclear respiratory factor 1	CCCCCGAGGACACTTCTTATGATG	GGCCGTTTCCGTTTCTTCCCTGTT
	Tfam	Transcription factor A, mitochondrial	GCTCTACACGCCCCTGGTTTCTGG	TCGCTGTAGTGCCTGCTGCTCCTG
	Pax7	Paired box7	AGGCCTTCGAGAGGACCCAC	CTGAACCAGACCTGGACGCG
	Pitx3	Pituitary homeobox 2	CGTTGAATGTCTCTTCTCCA	CTGGCCCTTATCTTTCTCAT
	Pitx2	Pituitary homeobox 3	TTCCCGTTCGCCTTCAACTCG	GAGCTGGGCGGTGAGAATACAGG
Differentiation	*Tnnt1*	Troponin T1	GTCCAACATGGGAGCTCATT	CAGACAGAATCCGGAGTTTCAT
	Sspn	Sarcospan	CTCAACGGGTCTGTGTCTTTAT	CAGTTTCTAGGGCTTTGGAGTAG
	Actn2	Actinin 2	TCCTGCTTCTACCATGCTTTC	CTTCCATCAGCCTCTCATTCTC
Enzymes	*Nox2*	NADPH oxidase 2	ACTCCTTGGAGCACTGG	GTTCCTGTCCAGTTGTCTTCG
	Nox4	NADPH oxidase 4	ACTTTTCA TTGGGCGTCCTC	AGAACTGGGTCCACAGCAGA
